# A Forest Wildlife Detection Algorithm Based on Improved YOLOv5s

**DOI:** 10.3390/ani13193134

**Published:** 2023-10-07

**Authors:** Wenhan Yang, Tianyu Liu, Ping Jiang, Aolin Qi, Lexing Deng, Zelong Liu, Yuchen He

**Affiliations:** College of Mechanical and Electrical Engineering, Hunan Agricultural University, Changsha 410128, China

**Keywords:** forest wildlife, data set annotation and augmentation, detection algorithm improvements, YOLOv5s, Swin Transformer, network convergence

## Abstract

**Simple Summary:**

We improved an algorithm for recognizing forest wildlife to increase the detection accuracy of wildlife in complex forest environments, and proposed a series of improvement schemes to address the high detection error and omission rate caused by the low contrast between the background and the target of the forest wildlife images captured by the trap camera, the serious occlusion and overlap, and the data imbalance, etc. A 16.8% improvement in the accuracy was finally achieved, which provides a new and feasible solution for the forest. This provides a new and feasible solution for forest identification and protection of wildlife provides a new feasible solution and idea.

**Abstract:**

A forest wildlife detection algorithm based on an improved YOLOv5s network model is proposed to advance forest wildlife monitoring and improve detection accuracy in complex forest environments. This research utilizes a data set from the Hunan Hupingshan National Nature Reserve in China, to which data augmentation and expansion methods are applied to extensively train the proposed model. To enhance the feature extraction ability of the proposed model, a weighted channel stitching method based on channel attention is introduced. The Swin Transformer module is combined with a CNN network to add a Self-Attention mechanism, thus improving the perceptual field for feature extraction. Furthermore, a new loss function (*DIOU_Loss*) and an adaptive class suppression loss (*L_BCE*) are adopted to accelerate the model’s convergence speed, reduce false detections in confusing categories, and increase its accuracy. When comparing our improved algorithm with the original YOLOv5s network model under the same experimental conditions and data set, significant improvements are observed, in particular, the mean average precision (*mAP*) is increased from 72.6% to 89.4%, comprising an accuracy improvement of 16.8%. Our improved algorithm also outperforms popular target detection algorithms, including YOLOv5s, YOLOv3, RetinaNet, and Faster-RCNN. Our proposed improvement measures can well address the challenges posed by the low contrast between background and targets, as well as occlusion and overlap, in forest wildlife images captured by trap cameras. These measures provide practical solutions for enhanced forest wildlife protection and facilitate efficient data acquisition.

## 1. Introduction

Traditional methods for tracking forest wildlife, such as direct searching with the eyes, collar tracking, sample line methods, and acoustic tracking using voice recorders, are still the main methods used to collect and record information about animals. However, these methods have certain limitations. Many zoologists believe that the collar tracking method is inconvenient to wildlife and can affect the health and movement of the animals [1]. Collars can have short- and long-term negative effects on wild animals. Plus, the act of capturing them is stressful and invasive. They can also modify the behavior of the collared animal. The acoustic tracking method is not applicable for all animals, mainly for burrowing mammals and actively vocalizing ones [2,3]. In addition, voice recorders must be installed correctly in order to register the animal. The sample line method consists of laying out sample lines for surveys, looking for traces of forest wildlife and signs of activity on the laid-out sample lines, and collecting relevant information for monitoring. All of the above methods would rely on statistic. However, this method is less efficient, requires a large investment in terms of human resources, and demands a high level of professional ability and proficiency from the monitoring individuals.

Trap cameras are widely used in the field of wildlife conservation. This method of acquiring image data has been used in scientific research and conservation efforts, such as wildlife monitoring, protection of endangered species, diversity surveying, and population density assessment. Camera traps are widely deployed by local wildlife conservation organizations and government departments to continuously acquire large amounts of image data from the field. For example, researchers at the Hunan Hupingshan National Nature Reserve in China have installed 150 infrared cameras for wildlife monitoring. Through these infrared cameras, researchers have observed the tracks and images of many rare animals. However, processing this image data and recognizing different forest wildlife species, as well as sorting out blank images (i.e., photos without wildlife), is a challenging task. As such, improving the efficiency of in-depth data mining and image processing and analysis approaches has become an urgent problem. Fortunately, state-of-the-art computer vision technologies and data-driven artificial intelligence techniques may play a role in solving this problem [4].

We propose an object detection algorithm based on improved YOLOv5, in order to verify the generalization ability, reliability, and robustness of the proposed algorithm, five rare forest wildlife species from the Hunan Hupingshan National Nature Reserve in China and five common forest wildlife species from North America were selected for experiments in this study. Rare animals such as *Ursus thibetanus*, *Lophura nycthemera*, *Prionailurus bengalensis*, *Macaca mulatta*, and *Elaphodus cephalophus* live in the Hunan Hupingshan National Nature Reserve, and are so scarce that they are all listed in the catalog of rare and protected animals. As it is very difficult to determine their habitats and collect survey information such as their population distribution manually, many localities have begun to use images from trap cameras to obtain information for studying these animals. Prior to deploying cameras, a 1 km^2^ or 1 ha grid is created for the entire monitoring plot, with pre-determined camera locations usually located at the center of each grid and printed on a map. We determine the path of camera deployment based on the walking routes in the surveillance area and place the camera locations at the current position (latitude and longitude) of the GPS navigator. The preset camera locations are found by GPS navigation and the location is used as a circle to find suitable camera placement locations within a radius of 20 m. After confirming the actual location of the camera, we re-record the GPS information of the camera location. During the layout, we try to choose paths where animals are active and there are nearby water sources. There should be no large leafy plants in front of the camera. We require fewer shrubs and grasses on the ground, especially during the plant growing season. Special attention should be paid to the growth of shrubs and grasses and to avoiding direct sunlight as much as possible. Some barriers can be erected, but care should be taken to preserve access for animal movement to ensure that animals have the maximum time before passing the camera. The camera is tied to a tree trunk approximately 0.5 m high with the camera head parallel to the ground.

Deep learning methods can effectively reduce the workload and improve the detection efficiency. To ensure the reliability of the algorithm, we also added five common forest wildlife species from North America to the data set of Hunan Hupingshan National Nature Reserve images, in order to verify the generalization ability and robustness of the algorithm.

Object detection is one of the fundamental tasks of computer vision, which involves detecting one or more classes of objects in image data. Deep learning methods are capable of extracting features and information about the target for network training through convolutional neural networks, and have become a popular solution for classifying automatic camera trap images [5,6,7]. However, field trap images contain information such as the location, size, and number of multiple targets, in addition to category information, due to their complex environmental context; thus, deep learning network methods utilizing target detection are better able to extract information and achieve improved detection results. The OverFeat [8], SPP-Net [9], RetinaNet [10], SSD [11] (Single Shot MultiBox Detector), RCNN series [12,13,14], and YOLO series [15,16,17,18] models are the most commonly used target detection networks. Target detection typically involves extracting feature vectors using wide and deep convolutional neural networks (CNNs). This process enables the prediction box to include both category and location coordinate information, significantly enhancing the recognition accuracy and regression rate for wildlife. With the regression rate indicating whether or not there were any missed detections in the detected images. Moreover, this method allows for precise target localization, facilitating the extraction of additional information, such as the number and behaviors of the identified targets. Due to the complexity of the wild environment, it is difficult to achieve good results when directly using the above target detection algorithms, and the accuracy rate needed to be further improved. After acquiring wildlife images, Chen et al. [19] developed two CNN structures. The proposed CNN-1 was used to classify six types of animals, including badgers (*Meles meles*), while CNN-2 combined the AlexNet network model with pre-training weights from the ImageNet data set, which shortened the training time and achieved better results in the detection of badgers from video streaming segments. Zhao et al. [20] designed a detection model called MobileNet-YOLO using a lightweight network structure MobileNet instead of the backbone feature extraction network of YOLOv4, deployed automatic camera traps in the field to capture wildlife images to form a data set, and also introduced migration learning [21] to solve the problems of insufficient training data and difficulty in network fitting. Migration learning improved the average accuracy, detection speed, and reduced the number of model parameters compared with YOLOv4.

The YOLOv5 target detection algorithm is characterized by a rapid detection speed and a lightweight model, which creates ideal conditions for improving the efficiency of image data processing. However, directly using the YOLOv5 network for detecting complex environments and forest wildlife with severe multi-obscuration overlap still results in high leakage and false detection rates. In the past few years, many new solutions have emerged in the field of computer vision, among which Vision Transformer (ViT) has performed outstandingly in many different computer vision tasks [22,23,24]. Therefore, it was considered worthwhile to investigate its combination with YOLOv5 to improve the performance of the network. The Transformer model was initially extensively applied in the field of Natural Language Processing (NLP) [25]. It extracts semantic information, sequence details, and distance information fusion from the input language and its context. By learning the relationships between components in a sequence, it constructs a global information interaction mechanism, characterized as a Self-Attention-based method; and is also known as a Self-Attention (SA)-based deep learning model. Vision Transformer [26] was the first Transformer model to be directly used in the field of visual processing, breaking the barriers between CV and NLP and greatly improving the accuracy of target detection. Better results were achieved when training it on the pre-trained model of a large-scale data set and then focusing it on a small data set for fine tuning. When utilizing the Vision Transformer model for feature extraction, it is common to divide the input image into specific segments prior to processing. Self-Attention [27] is employed to weigh and summarize the elements at various positions within the sequence to capture global information, resulting in each element within the entire sequence being calculated once. However, this approach has a high computational cost.

The Swin Transformer is a novel multi-layer visual Transformer backbone network [28], which borrows many design concepts—as well as prior knowledge—from convolutional neural networks, and uses a mechanism called Shifted Window to divide the input image into multiple chunks before interacting with the information between these chunks. This avoids the computational burden of processing the whole image at once. By introducing the Shifted Window Self-Attention mechanism, the input sequence is divided into several non-overlapping chunks, and the similarity between the elements in each chunk is calculated. This is different from the standard Self-Attention mechanism, which requires calculation of the similarity between all of the elements: the Shifted Window Self-Attention mechanism only needs to calculate the similarity between each element and the elements within a certain range around it, which avoids the need to compute too many similarities and thus improves the computational efficiency. The Swin Transformer has a surprising track record in a wide variety of computer vision tasks, including image categorization [29], target detection [30], and semantic segmentation [31]. Specifically, the Swin Transformer has demonstrated remarkable robustness and generalization ability, particularly when dealing with random local occlusion, severe occlusion of foreground objects, and interference from the background. Studies conducted on public data sets have indicated that the Swin Transformer outperforms other models in handling severely occluded objects [32]. However, using the Swin Transformer alone for target detection requires a lot of computational resources, as the encoder–decoder structure of the Swin Transformer is different from that of traditional CNNs, the acquisition of local information is not as strong as that of CNNs, and a certain amount of vanishing of the top gradient occurs. Therefore, we aimed to retain the advantages of both models as much as possible by combining the Swin Transformer module with the CNN-based YOLOv5s model, referring to other effective computer vision techniques to further improve the network, including introduction of the SENet channel attention mechanism and alteration of the loss function. The primary goal of this study is to use the improved SwinTR-Yolov5s to accurately and effectively detect wildlife in complex forest environments.

## 2. Materials and Methods

### 2.1. Data Acquisition and Pre-Processing

#### 2.1.1. Forest Wildlife Data Set

**Figure 1:**
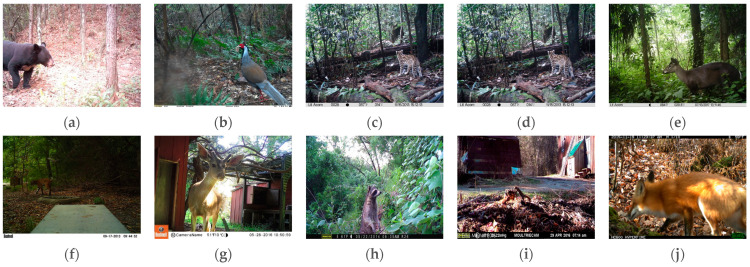
Forest wildlife data set example images: (**a**) *Ursus thibetanus*; (**b**) *Lophura nycthemera*; (**c**) *Prionailurus bengalensis*; (**d**) *Macaca mulatta*; (**e**) *Elaphodus cephalophus*; (**f**) *Lynx rufus*; (**g**) *Odocoileus hemionus*; (**h**) *Procyon lotor*; (**i**) *Tamiasciurus hudsonicus*; and (**j**) *Vulpes vulpes*.

The data sets used for the experiments in this paper were divided into two sets: data set 1 includes five rare forest wildlife species from the Hunan Hupingshan National Nature Reserve in northwestern Shimen County, China Hunan Province, China over the past five years; namely, *Ursus thibetanus* (Figure 1a), *Lophura nycthemera* (Figure 1b), *Prionailurus bengalensis* (Figure 1c), *Macaca mulatta* (Figure 1d), and *Elaphodus cephalophus* (Figure 1e). Data set 2 comprises the previous five categories of wildlife, as well as a screened subset of the 2019 iWildCam Wildlife Identification public data set filmed in North America [33]. This is an international competition data set for image detection, which has great validation significance for our model and is only used to further validate the generalization ability and completeness of the model, and ensure that the model still performs well on more types and complex data sets, which included *Lynx rufus* (Figure 1f), *Odocoileus hemionus* (Figure 1g), *Procyon lotor* (Figure 1h), *Tamiasciurus hudsonicus* (Figure 1i), and *Vulpes vulpes* (Figure 1j). As such, data set 2 was expanded to 10 forest wildlife species, as shown in Figure 1. These data sets had the following characteristics: (1) the image backgrounds are complex, including jungles, forests, bushes, rivers, and so on; (2) the images were taken at different locations at different times of the day, with large differences in light intensity and background; (3) some of the detected targets were group animals and, therefore, were densely arranged; and (4) some animals belonged to the same family and had small differences in appearance.

#### 2.1.2. Data Set Annotation and Augmentation

**Figure 2:**
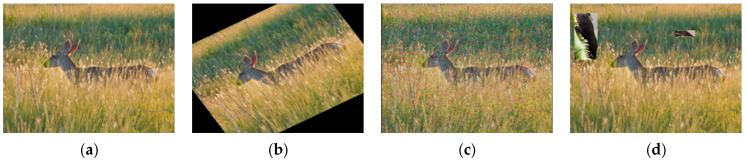
Illustrations of data set augmentation: (**a**) original image; (**b**) rotated image adjustment; (**c**) Gaussian blur/noise; and (**d**) image fusion.

As the network supports multi-resolution images for training, all images were scaled to the standard size of 640 × 640 during training. Thus, the data set did not have a uniform image pixel size, and the annotation was performed manually using the open-source tool Labelimg. After labeling all the original images (Figure 2a), data enhancement and augmentation were performed using computer vision techniques to further enrich the data set, including rotated image adjustment (Figure 2b), Gaussian blur/noise (Figure 2c), and image fusion (Figure 2d). Our image fusion method combined the advantages of both the Cutout and CutMix data enhancement methods. Cutout [34] uses fixed-size rectangular squares to randomly fill a region, masking the information in the filled part of the image, which helps to improve the generalization ability of the model. CutMix [35] was used to crop a part of an image and then fill the new one with a certain proportion. The real frame image, labeled with the cropped data set, was used for filling. Due to the influence of different pixels in the data set images, the cropped images were subjected to a normalized scaling operation. The images were synthesized according to the intersection and merger ratio (*IOU*), the filling area avoided the main information of the picture, and each image was randomly filled with two to four images to form a new data set. This method enriched and enhanced the information of the images, improving the generalization ability of the model and enhancing the detection effect, to a certain extent. In the process of data enhancement, the labeled key points and bounding boxes were converted accordingly, and the enriched data set was able to improve the detection performance of the network. As some images were poorly enhanced, after further screening, the final data set 1 was expanded to 5853 images and data set 2 was expanded to 21,300 images, which were divided into training (80%) and validation (20%) sets, as shown in Table 1, which were used to develop the forest wildlife detection model.

### 2.2. Experimental Conditions

In this paper, the training environment was the Linux version 18.04 operating system with an NVIDIA GeForce RTX 3080 GPU. The detection network was built based on the PyTorch deep learning framework, the Python development language version was 3.9, the compiled IDE was PyCharm, the CUDA version was 10.1, the Learning rate was set to 0.01, the Optimizer was Adam, the Batch Size was set to 8, and the number of Epochs was 200. The parameterization of the experiments is partly based on the original YOLOv5 optimal parameter selection and partly depends on the selection of the performance of the experimental platforms.

### 2.3. Forest Wildlife Detection Network

#### 2.3.1. YOLOv5

YOLO is a single-stage target detection algorithm that operates with the help of regression techniques, which create conditions for more efficient image processing. All of these factors create ideal conditions for improving the efficiency of image data processing. YOLOv5 not only improves the speed, volume, and accuracy compared to the previous generations of YOLO networks, but its code is also more concise and easier to understand, more convenient to use, and more convenient to deploy. YOLOv5 has four versions with the same network structure but with different widths and depths: YOLOv5s, YOLOv5m, YOLOv5l, and YOLOv5x. The Yolov5s network is the least deep in the Yolov5 family and has the smallest width of the feature map. The next 3 are deeper and wider on top of that. The network depth refers to the total number of layers of the network, and the network width refers to the number of convolution kernels in the convolutional layers, which is the number of channels (the third dimension of the feature map after convolution), which can be understood as the thickness of the network. Among these four versions, YOLOv5s has the smallest number of network parameters and calculations. Therefore, for efficient detection, the basic version chosen for this paper was YOLOv5s. The structure of YOLOv5s is divided into four components: Input, Backbone, Neck, and Position.

**Figure 3:**
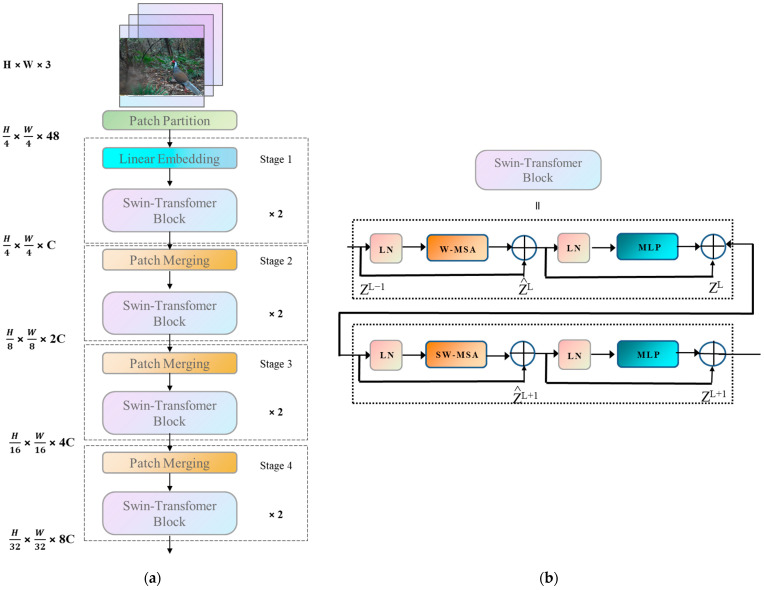
Structural diagram of Swin Transformer: (**a**) Overall architecture of the Swin Transformer; (**b**) two successive Swin Transformer blocks.

Input: The mosaic data enhancement method was used to randomly scale, crop, arrange, and splice the pictures, enhancing the detection of small targets and improving the training efficiency and robustness of the network. The effect of using mosaic data enhancement is shown in Figure 2. Adaptive image scaling was conducted to scale the original picture uniformly to a standard size and, based on the different aspect ratios of the different pictures, the black edges were adaptively added. The effect is shown in Figure 3, resulting in reduced computation and greatly improved speed of reasoning.

**Figure 4:**
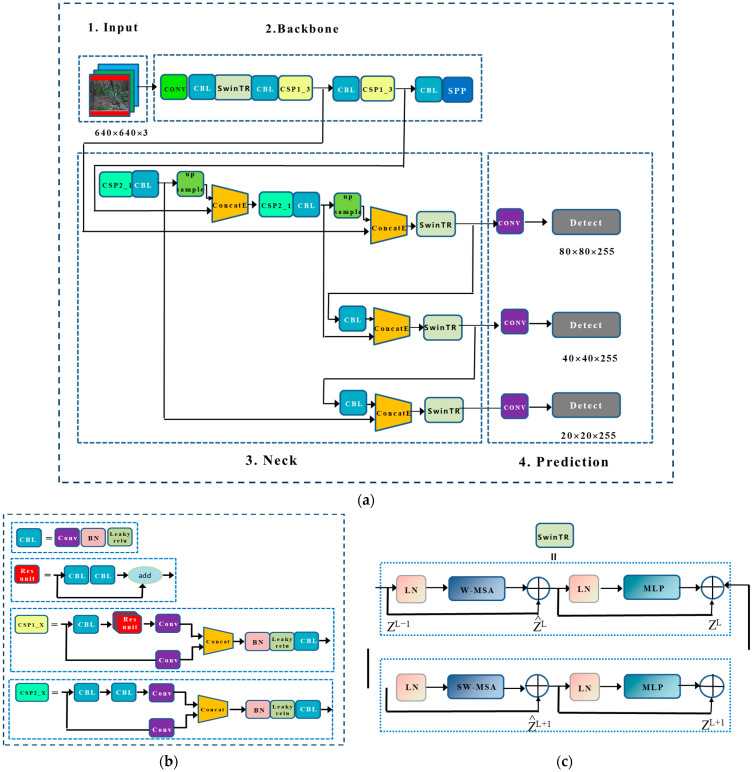

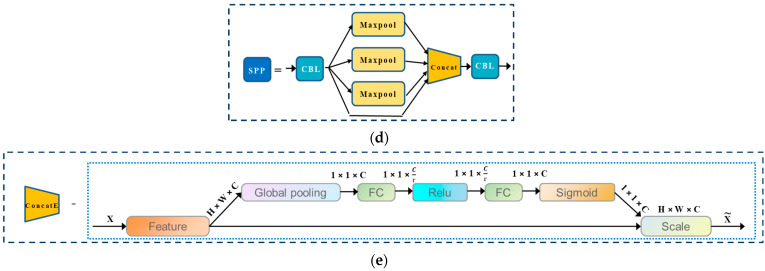
Overall architecture of the CNN–Swin Transformer Network: (**a**) optimized network structure diagram; (**b**) schematic diagram of CPS structure; (**c**) schematic diagram of Swin Transformer structure; (**d**) schematic diagram of SPP structure; (**e**) schematic diagram of ConcatE structure.

Backbone: The backbone network was used to extract feature maps from the input images with multiple convolutions and merging. A three-layer feature map was then generated in the backbone network in the sizes of 80 × 80, 40 × 40, and 20 × 20. In order to reduce the computational load of the model and to speed up the training process, YOLOv5 introduces the Focus module that slices and splices the image, which can first divide the input 3-channel image into four slices using the Slice operation. The four slices are connected using the Concat operation, and then the output feature mapping is generated using a convolutional layer. An illustration of the modules CBL, CSP1_X, residual components in the backbone network is given in Figure 4b, where CBL is a standard convolution module consisting of CONV, Batch Normalization (BN), and Sigmoid Linear Unit (SiLU) activation functions; CSP1_X is applied to the backbone part, X stands for X residual components, splitting the initial input into two branches, one branch passes through the CBL first, then through multiple residual structures, and then another convolution, the other branch is directly convolved, and then the two branches are concatenated, and then pass through the BN (normal distribution) layer, and then there is another activation to increase the residual structure, which increases the inverse layer to layer. This can increase the value of the gradient propagation between the layers to avoid the gradient disappearance due to deepening, so that more fine-grained features can be extracted and we do not have to worry about the network degradation, CSP2_X relative to CSP1_X, the only difference is that the CSP2_X will be Res unit replaced by CBL, mainly used in the Neck network. Finally, we use the spatial pyramid pooling (SPP) module to improve the sensory field by converting feature maps of arbitrary size into fixed-size feature vectors (Figure 4d). The feature map is first output through a CONV layer with a kernel size of 1 × 1. It is then connected to the output of the feature map subsampled by three parallel maximal pooling layers, followed by the CONV layer to output the final feature map.

Neck network: The Neck network contains a series of feature fusion layers that blend and combine image features. All feature maps of different sizes generated by the backbone network are fused to obtain more contextual information and reduce information loss. In the merging process, the feature pyramid structure of the Feature Pyramid Network (FPN) and Path Aggregation Network (PANet) is used, and the FPN structure is utilized to transfer the strong features from the top to the bottom feature map. Meanwhile, PANet is used to transfer the strong localization features from the lower feature map to the higher feature map. In conclusion, the combined use of FPN and PANet enhances the feature fusion capability of the Neck network. It consists of three detection layers corresponding to output feature maps of 80 × 80, 40 × 40, and 20 × 20 for detecting objects in the input image. Each detection layer can ultimately output 21-channel vectors, which are then generated and labeled with predicted bounding boxes and categories of the target in the original input image for final detection.

Output side: Non-Maximum Suppression (NMS) was used in the post-processing process for multi-target screening, with *GIOU* as the loss function.

#### 2.3.2. Swin Transformer

A compact version of the Swin Transformer [28] architecture is illustrated in Figure 3a. It first splits an input RGB image into non-overlapping patches using a patch splitting module, such as ViT. Each patch is treated as a token, whose features are set as the concatenation of raw pixel values in the RGB image. In this process, a patch size of 4 × 4 is used. Several Transformer blocks with modified Self-Attention computation (Swin Transformer blocks) are applied to these patch tokens. The structure of the Swin Transformer blocks is also shown in Figure 3. Unlike the convolutional neural network with residual computation of YOLOv5s—which is first normalized by a normalization layer (Batch Normalization, LN)—in the Windows Multi-Head Self-Attention calculation module (Windows Multi-Head Self-Attention, W-MSA), the image is divided into four non-overlapping window regions, and each window calculates the Self-Attention independently. Then, after residual computation and renormalization, it enters into a Multi-layer Perceptron. The Multi-layer Perceptron (MLP) is a neural network layer using non-linear activation function, which carries out non-linear mapping and accelerates the fitting of the network. Finally, another residual computation is carried out to obtain the new output features and enter the second round of sliding window computation. The second round has the same structure as the first round, except that a sliding window operation is performed in the sliding window multi-head Self-Attention layer.

#### 2.3.3. SENet Channel Attention Mechanism

In order to maximize the retention of local information while condensing the fusion information, the channel attention mechanism SENet was proposed by Hu et al. [36]. It learns the feature weights, obtains the importance degree of each feature map, and assigns a weight value to each feature channel according to the degree of importance, which enhances the effectiveness of the information and suppresses irrelevant information, allowing the model to achieve better results. In order to easily and intuitively compare it with the original algorithm’s Concat module, we denote the SE module by ConcatE in this paper. The ConcatE module is depicted in Figure 4e, in which H is the height (height), W is the width (width), and C is the number of channels. First, the Squeeze operation is carried out on the convolutionally obtained feature map, which uses global average pooling to compress H × W × C into 1 × 1 × C. After H × W is compressed into one dimension, it is equivalent to a one-dimensional parameter to obtain the global vision of H × W, and the perceptive region is wider. Then, the Excitation operation is performed on the global features and two fully connected layers are used to learn the correlations between channels. The first FC layer has the role of dimensionality reduction, then ReLU activation is used, while the final fully connected layer restores the original dimensionality. The obtained features are multiplied by the original feature map to obtain the final features. Essentially, the SE module introduces an attention mechanism to the channel dimension, which allows the model to pay more attention to the most informative channel features and suppress unimportant channel features.

#### 2.3.4. Integration of Swin Transformer and SENet-YOLOv5

First, in order to utilize the advantages of both the Swin Transformer and YOLOv5, we replaced the first CSP_1 layer of the original YOLOv5 backbone network and the CSP_2 layer located in the three layers of the Neck network to extract multi-layer features with the Swin Transformer module, while retaining the rest of the CNN-based CBL and CSP layers. YOLOv5, as a typical CNN, has excellent local sensing ability but lacks global modeling capacity [37]. The Swin Transformer is based on the multi-head Self-Attention mechanism, which can be used to capture long-distance dependencies and thus obtain global information. Therefore, in our proposed method, by combining YOLOv5s and the Swin Transformer, the new structure leverages both of their advantages to retain global and local features. Second, in order to further optimize the structure, we introduce the ConcatE module of SENet based on the attention mechanism, in order to learn the splicing process of the global and local features in a weighted manner while retaining features that have an important influence on the detection results. Our proposed fusion network is shown in Figure 4, which is more effective for wildlife detection when considering the complex environment of forests.

#### 2.3.5. Loss Function Improvement

The loss function of YOLOv5 generally contains the prediction error of the prediction frame, the prediction frame confidence error, and the target category error. The loss function of the prediction error of the prediction frame adopts the *GIOU* loss function, which is calculated as follows:

(1) $$GIOU_{Loss}=IOU-\frac{C-\left(A\cup B\right)}{C},$$

where A and B represent the prediction frame and the real frame, respectively, and C is the minimum outsourcing frame that contains both A and B. *GIOU* solves the problem that the gradient of the *IOU* loss function cannot be calculated and adds a penalty term to the minimum outsourcing area, but it still cannot resolve the problem of slow convergence, increased iteration times, and increased computation brought about by the fact that the initial prediction frame does not intersect with the real frame.

The YOLOv5 prediction frame confidence error and target category error loss are both calculated using the two-cross entropy loss function with the following formula:

(2) $$BCE\left(x_{i}\right)=-\sum_{i=1}^{C}Log\left({\hat{C}}_{i}\right),$$

where ${\hat{C}}_{i}$ is the predicted value of traversing all categories, which takes a value between 0 and 1. The two-cross entropy loss function is unfavorable for the classification of positive and negative samples, to a certain extent, and positive and negative samples are generally unbalanced. In this case, when a network is being trained, the focus of the training attention tends to the category with the most samples, and the excessive inhibitory gradient generated by the dominant (head) category will seriously hinder the detection results on the other (tail) categories.

To address the above problems, the improved loss function given in Equations (3)–(5) is used to first improve the prediction error of the algorithm prediction frame using *DIOU_Loss* [38], in order to introduce a new penalty term for geometric factors, which not only solves the non-overlapping problem, but also speeds up convergence.

(3) $$DIOU\text{\_}Loss=1-IOU+\frac{\rho^{2}\left(b,b^{gt}\right)}{c^{2}}$$

where b and $b^{gt}$ are the centroids of the prediction and target boxes, respectively; $\rho^{2}$ is the square of the Euclidean distance calculated between the two centroids; and $c^{2}$ is the square of the diagonal length of the smallest rectangular box containing the prediction and target boxes.

Second, we use a new adaptive class suppression loss to protect the training of tail data [39] and adaptively select the classes to be suppressed based on the learning state.

(4) $$L_{BCE\left(x_{i}\right)}=-\sum_{i=1}^{c}\omega_{i\;}Log\left({\hat{C}}_{i}\right),$$

(5) $$\omega_{i}=\left\{\begin{array}{cc} 1 & if\;i=k\\ 1, & if\;i\neq k\;and\;c_{i}\geq \alpha \\ 0, & if\;i\neq k\;and\;c_{i}<\alpha \end{array}\right.$$

As shown in Equations (4) and (5), we multiply the weighting term $\omega_{i\;}$ with the loss term $-Log\left({\hat{C}}_{i}\right)$ of category i and traversed all categories, where $\omega_{i}$ takes a value of 1 if the detection category i belongs to the true category k. For the other categories i (i≠k), we judge whether the confidence of the outputs of this category exceeded the threshold α. We utilize the output confidence $c_{i}$ as a signal to determine whether to suppress category i. If a category has $c_{i}$ greater than or equal to the threshold α, this implies that the network is confusing the categories i and k. Therefore, we set $\omega_{i\;}$ to 1 for discriminative learning and set $\omega_{i\;}$ to 0 to go against it, in order to avoid unwanted negative inhibition.

## 3. Results

### 3.1. Evaluation Criteria

In order to evaluate the effectiveness of our proposed method in terms of detection in forest wildlife trap images, the accuracy (Precision; P), regression rate (Recall; R), mean average precision (*mAP*), and detection speed (Frames Per Second; FPS) were chosen as evaluation indicators. The AP is calculated as the area under the precision–recall curve, while the *mAP* is obtained by averaging the AP for all detection categories; the larger the *mAP*, the better the model detection effect. For this paper, we adopted the *mAP* (50%); that is, the threshold was 0.5 and a confidence level exceeding 0.5 was the target. The *P*, *R*, and *mAP* indices were calculated as follows:

(6) $$P=\frac{TP}{TP+FP^{\;}},$$

(7) $$R=\frac{TP}{TP+FN},$$

(8) $$mAP=\frac{\sum_{i=0}^{N-1}\int_{0}^{1}P\left(R\right)dR}{N},$$

where *TP* denotes the number of correctly recognized images, *FP* denotes the number of incorrectly recognized images, and *FN* denotes the number of missed images.

### 3.2. Results of Forest Wildlife Detection Experiments

#### 3.2.1. Ablation Study

We designed a series of ablation experiments to validate the performance of the proposed algorithm and tested it on data set 1 with and without data augmentation, the ConcatE structure, the SwinTR module, *DIOU_Loss* and *L_BCE*. All networks were pre-trained models trained on the COCO data set [40] using the officially provided YOLOv5s network with the same hyper-parameter settings. The training, validation, and testing data sets were obtained from the data set described in this paper, in order to control the variables and ensure the validity of the results.

For the first group of tests, we used the data set before data enhancement and modeled the YOLOv5s network; the second group used the data set after data enhancement and modeled the YOLOv5s network as well; the third group added the ConcatE structure in addition to the conditions of the second group; the fourth group introduced the SwinTR module, also on top of the second group; the fifth group added both the ConcatE and the SwinTR module; and the sixth group replaced the new loss functions *DIOU_Loss* and *L_BCE* on the basis of the fifth group. The test results for all networks are provided in Table 2.

From Table 2, it can be seen that, when the data were enhanced, the degree of attention to the important feature information of wildlife through the attention module was improved, combining the advantages of the global attention associated to the SwinTR module with those of convolutional kernel extraction of features by the CNN in order to improve the performance of the network in terms of extracting feature information. Furthermore, using the loss functions *DIOU_Loss* and *L_BCE* improved the accuracy and speed of the prediction frame regression and reduced detection errors. Compared to the initial network, the final proposed method improved the *mAP* value by 16.8%.

**Figure 5:**
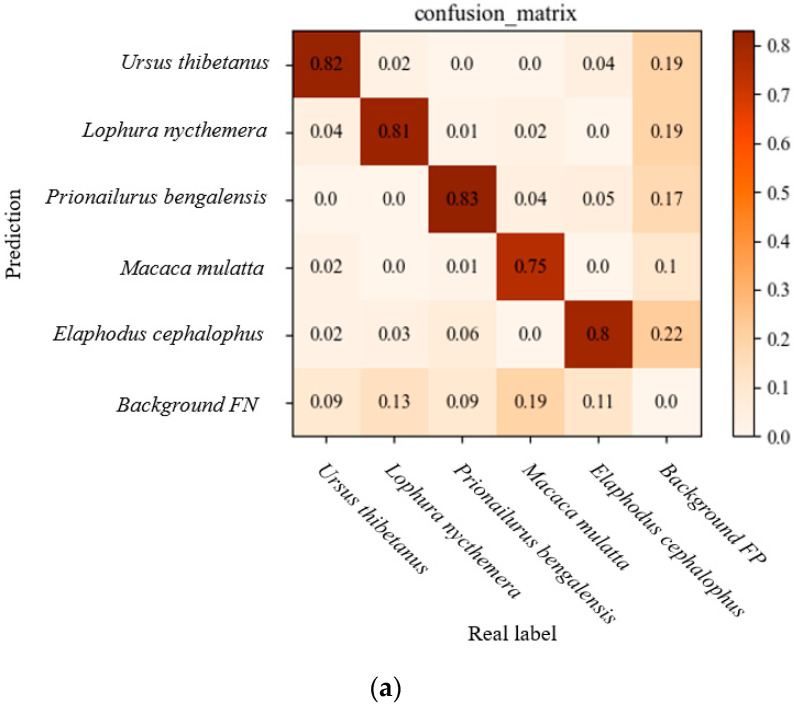

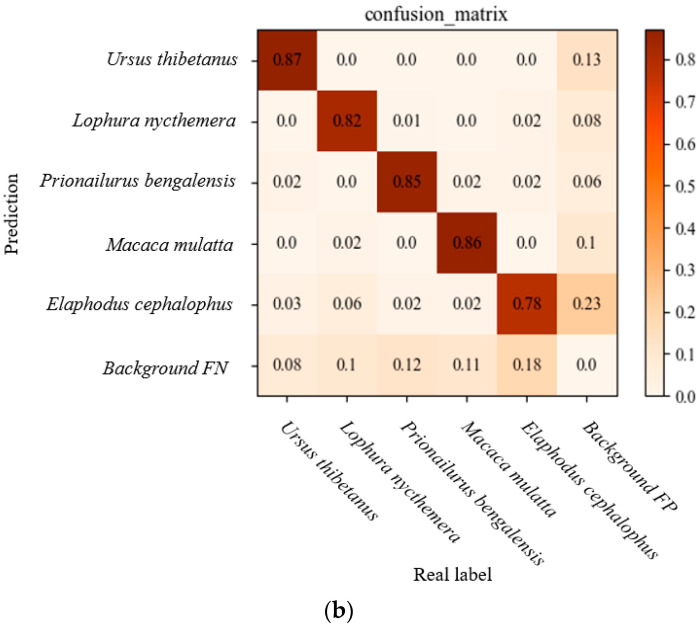
Confusion matrix comparison. (**a**) pre-improvement; (**b**) post-improvement.

We verified the effect of modifying the loss functions on the detection results by further observing the changes in the confusion matrix, as shown in Figure 5a,b; the FP of background refers to the probability of mistakenly treating rare wild animals that were not originally background as background, resulting in missed detection of the corresponding rare wild animals, while the FN of background refers to the probability of identifying the background as the corresponding wild animals, falsely detecting objects that were not originally present, and causing false detection. We found that the model obtained after improving the loss function reduced the misdetection rate of different species of wildlife, and the environmental misdetections and omissions under complex backgrounds were also optimized, to a certain extent. Compared with the improvement of the loss function before and after, we found that the model significantly improved the detection accuracy of *Ursus thibetanus*, *Lophura nycthemera*, *Prionailurus bengalensis*, and *Macaca mulatta*, with only *Elaphodus cephalophus* slightly reduced. We speculate that this is due to the fact that the environment in which *Elaphodus cephalophus* is active is similar to its own color, making it difficult to effectively distinguish the background. Overall, the improvement of the loss function has indeed optimized the false detection rate of the model in distinguishing multiple wild animals, which can to some extent solve the problem of low accuracy caused by data imbalance.

Next, we compared our improved model with other popular detection models. The results are given in Table 3. We found that Faster-R-CNN, as a two-stage target detection algorithm, had a certain advantage in terms of detection accuracy but, due to the need to extract feature vectors in the feature region in both the training stage and detection stage, it wasted a significant amount of memory and time, required a lot of hardware equipment, had a slow detection speed, and cannot realize real-time detection, making it unsuitable for further research and applications. Due to the diverse morphology and complex background of wildlife in the data set, the detection accuracy of the RetinaNet method was not high, and the YOLO series of methods—which are lightweight and can achieve rapid detection—had problems related to misdetection and omission under the complex backgrounds. Our improved model obtained a lower misdetection rate and false detection rate while making substantial improvements in accuracy. It also inherited the advantages of the YOLOv5s model, with the best overall performance.

**Figure 6:**
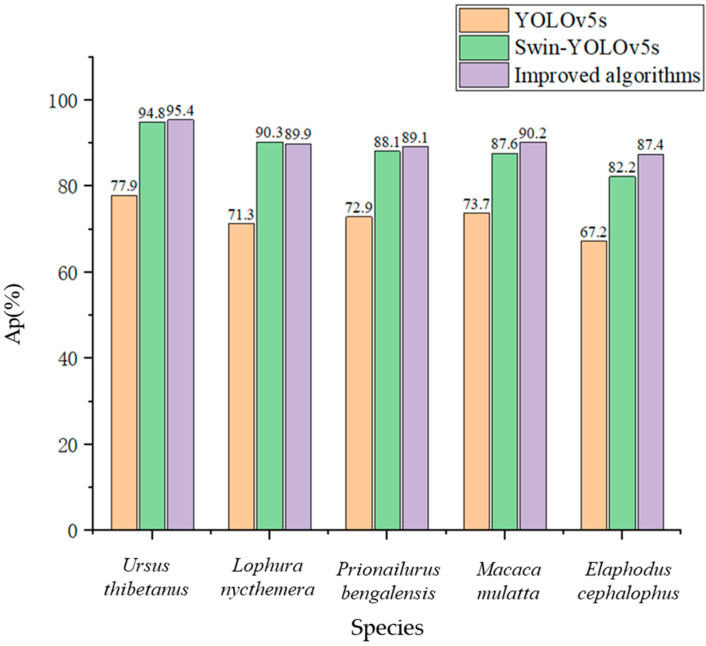
Comparison of three structural models on data set 1.

**Figure 7:**
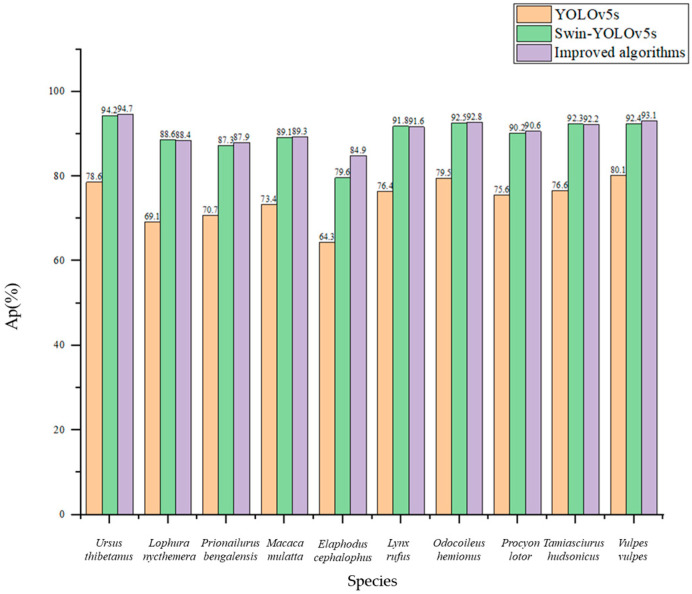
Comparison of three structural models on data set 2.

After determining that our model outperforms other popular models, in order to verify that it also outperforms the convolutional feature extraction model and the Swin Transformer feature extraction model individually, we further compared the initial YOLOv5 model, the Swin-YOLOv5s model, with all CSP convolutional modules replaced with SwinTR modules, and our fusion improved algorithm on data set 1 and data set 2. The test results are shown in Figure 6 and Figure 7. It can be found that the improved model has a significant detection improvement effect on all categories of wild animals, and the bird *Lophura nycthemera* is not significantly different from other mammals. The model has the best improvement effect on *Elaphodus cephalophus* detection in mammals, as the false detection rate brought by *Tamiasciurus hudsonicus* from the same family of animals has been controlled to some extent. However, due to its minimal training data, it is still difficult to achieve high detection accuracy. Our improved model has the best performance in detecting larger wild animals. However, the performance improvement is greater for detecting small- and medium-sized targets, this is thanks to the multi-scale fusion mechanism of the global attention mechanism of the Swin Transformer module. In addition, the detection speed of the model decreased accordingly as more Swin Transformer modules were used, due to the greater computational requirements of the Swin Transformer modules.

#### 3.2.2. Test Results

**Figure 8:**
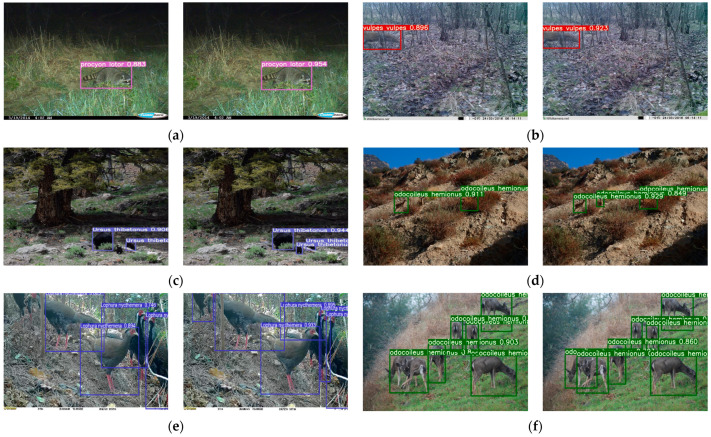
Test combination comparison: (**a**) normal target detection; (**b**) incomplete object detection; (**c**) small target detection; (**d**) small targets that are difficult to distinguish between target and background; (**e**) overlapping target detection in case of animal and background similarity; (**f**) overlapping target detection in situations where animals are easily distinguishable from the background.

Figure 8 shows some of the test results based on images from the test set of data set 2. The left side of Figure 8a–f shows the detection results for the initial model of YOLOv5s, while the right side shows the detection results for the improved algorithm proposed in this paper. From the detection results shown in Figure 8a,b, it can be observed that both models had good capabilities when processing clear, medium-sized, and incompletely captured images; however, our model performed better in terms of confidence. Further observation of Figure 8c reveals that the initial model YOLOv5s showed leakage regarding the detection of smaller target; in Figure 8d, leakage also occurred for the detection of small targets where it was difficult to distinguish between the target and background. However, our improved model successfully avoided these problems. In addition, in Figure 8e,f, the initial model appeared to suffer from inaccurate localization and missed detection when the targets had high overlap, while our improved algorithmic model solved this problem.

## 4. Discussion

To address the problem of insufficient detection accuracy when using existing methods for the detection of forest wildlife in complex environments, we proposed an improved model. To evaluate the performance of this model, we compared it with other commonly used detectors, including YOLOv5, YOLOv3, RetinaNet, and Faster-RCNN. By comparing the experimental results, we were able to draw the following conclusions:

1. Animal detection based on the Swin Transformer model has good results [41,42]. In contrast, the improved method we propose in this paper is based on the original YOLOv5 network model and takes some steps to improving the training of the model. First, we use our proposed data enhancement method and some enhancement methods to enhance the richness of the data set. Second, we introduced the idea of channel-based attention by replacing the original Concat with a weighted channel splicing method (denoted as ConcatE), which increases the number of channel layers for key feature information and improves the attention to important channel information. In addition, we found the optimal backbone network structure suitable for this data set through comparative experiments, and we used the Swin Transformer module to replace the CSP_1 layer in the YOLOv5 backbone network and the CSP_2 layer in the Neck network, while retaining the other CNN-based CBL and CSP layers, thus taking advantage of convolutional, attentional, and Self-Attentional mechanisms. To address the non-overlap problem, we employ a new loss function (*DIOU_Loss*) to speed up the convergence of the model and introduce an adaptive class suppression loss (*L_BCE*) to suppress false detection of confusing classes and ensure the accuracy of the tail data. Ensuring the accuracy of detection between animal species with high similarity levels. By analyzing the confusion matrix, we find that *L_BCE* further reduces the impact of data imbalance on the detection results and improves the detection accuracy. The experimental results demonstrate the sophistication of our improved model with an accuracy of 90.2%, a recall of 83.3%, and a *mAP* of 89.4%.
2. Based on the experimental results, we observed that the difference between the detection results of the models before and after the proposed improvements on two data sets with different data volumes was relatively small, and all of the improved methods achieved significant improvements. In particular, the experimental results on data set 1 indicated that our improved algorithm model improved the *mAP* metric by 16.8%, 20%, 16.9%, and 10.5% when compared to the YOLOv5s, YOLOv3, RetinaNet, and Faster-RCNN methods, respectively. These results indicated that our improvements were very effective in enhancing the detection performance of the proposed model. In addition, our improved algorithm is well suited for edge deployment and embedded development with the help of some control algorithms [43] and hardware device [44], as the inference speed of the model ensures the feasibility of real-time detection.
3. Our model effectively solves some of the problems of omission and false detection that occur during the detection process in complex environments. The difference between the detection results of ten types of forest wildlife before and after the improvement of the two models is not significant, and the effect of data collection area on wildlife detection results is also not significant. Although the best test results were obtained from the *Ursus thibetanus* in the Hupingshan National Nature Reserve, rather than the *Odocoileus hemionus* with the most abundant training data, with a *mAP* of 94.7, the detection accuracy of other wild animals in Hupingshan was lower than that of North American wild animals with richer training data. These results are reasonable. *Ursus thibetanus* are characterized by high discrimination, large feature differences, large size, and relatively sufficient training data. Therefore, the biggest factor affecting the detection results in the first place remains the training data, which is closely related to the amount and diversity of data. Secondly the single-stage detection algorithm based on regression thinking is better at detecting large-sized targets than small ones, and we optimize the detection ability for small targets. In addition, the probability of false detection is greater for conspecifics with high feature similarity, and we also propose an improvement method for this point, which effectively solves the problem of maintaining a high level of detection accuracy when detecting animal species with high similarity.
4. Although we undertook some work to improve the algorithmic model, there are still some shortcomings. Specifically, we observed some contradictions between the complexity of the network structure and the model detection performance. In order to balance the model detection performance and FPS performance, we made certain tradeoffs. We employed multi-scale feature fusion and global feature extraction, which increased the computational effort and slowed down inference. Although we lost some of the original inference speed, to a certain extent, this improved the model’s detection of difficult targets. The current GPU acceleration optimization of the Transformer model is not sufficient, which limits the inference speed. However, with the optimization of GPU hardware support and improvement of the model structure in the future, the speed of Transformer-based models is expected to further improve. In addition, we intend to work on improving the proposed algorithm through the use of more efficient strategies [45] to reduce the impact on FPS in future research.

## 5. Conclusions

In this paper, we design a Swin Transformer-YOLOv5 detection network for forest wildlife based on YOLOv5s. We use a combination of some current techniques in computer vision, and by testing on the data set, the improved algorithm has better overall performance compared with other commonly used target detection models, achieving a *mAP* of 89.4% while the FPS is 40, and the parameter size is only 15.2 MB, which realizes the high precision and high efficiency remote target detection of wildlife images in the complex environment of the forest, and has the ability of real-time detection, which means that it is a target detection algorithm with practical value, and it can provide a convenient and effective method of wildlife initial detection for forest wildlife monitors, and promote the monitoring and protection of the forest wildlife action work, and we also hope that this paper can be helpful to more developers and researchers dealing with forest wildlife classification.

## Figures and Tables

**Table 1:** Data set composition.

Area	Species	Number of Images	Number of Adjustments of Rotation	Number of Gaussian Blur/Noise	Number of Image Fusions	Total
	*Ursus thibetanus*	525	525	453	525	
	*Lophura* *nycthemera*	400	400	362	400	
China	*Prionailurus bengalensis*	327	327	293	327	5853
	*Macaca mulatta*	162	162	131	162	
	*Elaphodus cephalophus*	93	93	93	93	
North America	*Lynx rufus*	647	647	621	647	
	*Odocoileus hemionus*	1065	1065	956	1065	
	*Procyon lotor*	655	655	611	655	15,447
	*Tamiasciurus hudsonicus*	804	804	761	804	
	*Vulpes vulpes*	758	758	711	758	

**Table 2:** Results of ablation experiments.

Group	Model	Average Accuracy (%)	Average Recall (%)	*mAP*@0.5 (%)	Detection Speed (FPS)
1	YOLOv5s	82.2	63.9	72.6	53
2	YOLOv5s + Data Augmentation	85.4	69.5	76.5	53
3	YOLOv5s + Data Augmentation + ConcatE	87.4	72.8	78.4	53
4	YOLOv5s + Data Augmentation + Swin T	89.4	74.6	85.5	41
5	YOLOv5s + Data Augmentation + ConcatE + Swin T	90.5	79.5	87.7	40
6	YOLOv5s+ Data Augmentation + ConcatE + Swin T + *DIOU_Loss* + *L_BCE*	90.2	83.3	89.4	40

**Table 3:** Performance comparison of different detection methods.

Model	*mAP*@0.5 (%)	Detection Speed (FPS)	Model Size (MB)
YOLOv5s	72.6	53	14.6
YOLOv3	69.4	41	240.8
RetinaNet	72.5	49	49.3
Faster-RCNN	78.9	34	112.6
Improved algorithm	89.4	40	15.2

## Data Availability

Not applicable.

## References

[1] Schneider T.C., Kowalczyk R., Köhler M. (2013). Resting site selection by large herbivores–The case of European bison (*Bison bonasus*) in Białowieza Primeval Forest. Mamm. Biol..

[2] Noad M.J., Cato D.H., Stokes M.D. Acoustic Tracking of Humpback Whales: Measuring Interactions with the Acoustic Environment. Proceedings of the Acoustics.

[3] Andreychev A.V. (2019). Daily and seasonal feeding activity of the greater mole-rat (*Spalax microphthalmus*, Rodentia, Spalacidae). Biol. Bull..

[4] Zou Z., Chen K., Shi Z., Guo Y., Ye J. (2023). Object Detection in 20 Years: A Survey.

[5] Chen G., Han T.X., He Z., Kays R., Forrester T. (2014). Deep convolutional neural network-based species recognition for wild animal monitoring. 2014 IEEE International Conference on Image Processing (ICIP).

[6] Villa A.G., Salazar A., Vargas F. (2017). Towards automatic wild animal monitoring: Identification of animal species in camera-trap images using very deep convolutional neural networks. Ecol. Inform..

[7] Norouzzadeh M.S., Nguyen A., Kosmala M., Swanson A., Palmer M.S., Packer C., Clune J. (2018). Automatically identifying, counting, and describing wild animals in camera-trap images with deep learning. Proc. Natl. Acad. Sci. USA.

[8] Sermanet P., Eigen D., Zhang X., Mathieu M., Fergus R., Lecun Y. (2013). Overfeat: Integrated Recognition, Localization and Detection using Convolutional Networks. arXiv.

[9] He K., Zhang X., Ren S., Sun J. (2015). Spatial pyramid pooling in deep convolutional networks for visual recognition. IEEE Trans. Pattern Anal. Mach. Intell..

[10] Wei F., Sun X., Li H., Wang J., Lin S. (2020). Point-set anchors for object detection, instance segmentation and pose estimation. Computer Vision–ECCV 2020: 16th European Conference, Glasgow, UK, 23–28 August 2020, Proceedings, Part X 16.

[11] Liu W., Anguelov D., Erhan D., Szegedy C., Reed S., Fu C., Berg A.C. (2016). Ssd: Single shot multibox detector. Computer Vision–ECCV 2016: 14th European Conference, Amsterdam, The Netherlands, 11–14 October 2016, Proceedings, Part I 14.

[12] Girshick R., Donahue J., Darrell T., Malik J. Rich Feature Hierarchies for Accurate Object Detection and Semantic Segmentation. Proceedings of the IEEE Conference on Computer Vision and Pattern Recognition.

[13] Girshick R. Fast r-Cnn. Proceedings of the IEEE International Conference on Computer Vision.

[14] Ren S., He K., Girshick R., Sun J. (2017). Faster r-Cnn: Towards Real-Time Object Detection with Region Proposal Networks. IEEE Trans. Pattern Anal. Mach. Intell..

[15] Redmon J., Divvala S., Girshick R., Farhadi A. You Only Look Once: Unified, Real-Time Object Detection. Proceedings of the IEEE Conference on Computer Vision and Pattern Recognition.

[16] Redmon J., Farhadi A. YOLO9000: Better, Faster, Stronger. Proceedings of the IEEE Conference on Computer Vision and Pattern Recognition.

[17] Redmon J., Farhadi A. (2018). Yolov3: An Incremental Improvement. arXiv.

[18] Li H., Jiang F., Guo F., Meng W. (2022). A real-time detection method of safety hazards in transmission lines based on YOLOv5s. International Conference on Artificial Intelligence and Intelligent Information Processing (AIIIP 2022).

[19] Chen R., Little R., Mihaylova L., Delahay R., Cox R. (2019). Wildlife Surveillance using Deep Learning Methods. Ecol. Evol..

[20] Zhao T., Yi X., Zeng Z., Feng T. (2021). MobileNet-Yolo based wildlife detection model: A case study in Yunnan Tongbiguan Nature Reserve, China. J. Intell. Fuzzy Syst..

[21] Pan S., Yang Q. (2009). A survey on transfer learning. Transactions on knowledge and data engineering. IEEE Trans. Knowl. Data Eng..

[22] Khan S., Naseer M., Hayat M., Zamir S.W., Khan F.S., Shah M. (2022). Transformers in vision: A survey. ACM Comput. Surv. (CSUR).

[23] Han K., Wang Y., Chen H., Chen X., Guo J., Liu Z., Tang Y., Xiao A., Xu C., Xu Y. (2022). A Survey on Vision Transformer. IEEE Trans. Pattern Anal. Mach. Intell..

[24] Li Y., Mao H., Girshick R., He K. (2022). Exploring plain vision transformer backbones for object detection. European Conference on Computer Vision.

[25] Bahdanau D., Cho K., Bengio Y. (2014). Neural Machine Translation by Jointly Learning to Align and Translate. arXiv.

[26] Dosovitskiy A., Beyer L., Kolesnikov A., Weissenborn D., Zhai X., Unterthiner T., Dehghani M., Minderer M., Heigold G., Gelly S. (2020). An image is worth 16 × 16 words: Transformers for image recognition at scale. arXiv.

[27] Vaswani A., Shazeer N., Parmar N. (2017). Attention is all you need. arXiv.

[28] Liu Z., Lin Y., Cao Y., Hu H., Wei Y., Zhang Z., Lin S., Guo B. Swin Transformer: Hierarchical Vision Transformer using Shifted Windows. Proceedings of the IEEE/CVF International Conference on Computer Vision.

[29] Jannat F., Willis A.R. (2022). Improving classification of remotely sensed images with the Swin Transformer. SoutheastCon 2022.

[30] Liu Z., Tan Y., He Q., Xiao Y. (2021). SwinNet: Swin Transformer drives edge-aware RGB-D and RGB-T salient object detection. IEEE Trans. Circuits Syst. Video Technol..

[31] Hatamizadeh A., Nath V., Tang Y., Yang D., Roth H.R., Xu D. (2021). Swin unetr: Swin Transformers for semantic segmentation of brain tumors in mri images. International MICCAI Brainlesion Workshop.

[32] Naseer M.M., Ranasinghe K., Khan S.H., Hayat M., Shahbaz Khan F., Yang M. (2021). Intriguing properties of vision transformers. Adv. Neural Inf. Process. Syst..

[33] Beery S., Morris D., Perona P. (2019). The iWildCam 2019 Challenge Dataset. arXiv.

[34] Devries T., Taylor G.W. (2017). Improved regularization of convolutional neural networks with cutout. arXiv.

[35] Yun S., Han D., Oh S.J., Chun S., Choe J., Yoo Y. Cutmix: Regularization Strategy to Train Strong Classifiers with Localizable Features. Proceedings of the IEEE/CVF International Conference on Computer Vision.

[36] Hu J., Shen L., Sun G. Squeeze-and-Excitation Networks. Proceedings of the IEEE Conference on Computer Vision and Pattern Recognition.

[37] Li T., Wang J., Zhang T. (2022). L-DETR: A Light-Weight Detector for End-to-End Object Detection with Transformers. IEEE Access.

[38] Zheng Z., Wang P., Liu W., Li J., Ye R., Ren D. Distance-IoU Loss: Faster and Better Learning for Bounding Box Regression. Proceedings of the AAAI Conference on Artificial Intelligence.

[39] Wang T., Zhu Y., Zhao C., Zeng W., Wang J., Tang M. Adaptive Class Suppression Loss for Long-Tail Object Detection. Proceedings of the IEEE/CVF Conference on Computer Vision and Pattern Recognition.

[40] Lin T., Maire M., Belongie S., Hays J., Perona P., Ramanan D., Dollár P., Zitnick C.L. (2014). Microsoft coco: Common objects in context. Computer Vision–ECCV 2014: 13th European Conference, Zurich, Switzerland, 6–12 September 2014, Proceedings, Part V 13.

[41] Agilandeeswari L., Meena S. (2023). Swin transformer based contrastive self-supervised learning for animal detection and classification. Multimed. Tools Appl..

[42] Gu T., Min R. A Swin Transformer based Framework for Shape Recognition. Proceedings of the 2022 14th International Conference on Machine Learning and Computing (ICMLC).

[43] Deng L., Liu T., Jiang P., Xie F., Zhou J., Yang W., Qi A. (2023). Design of an Adaptive Algorithm for Feeding Volume–Traveling Speed Coupling Systems of Rice Harvesters in Southern China. Appl. Sci..

[44] Deng L., Liu T., Jiang P., Qi A., He Y., Li Y., Yang M., Deng X. (2023). Design and Testing of Bionic-Feature-Based 3D-Printed Flexible End-Effectors for Picking Horn Peppers. Agronomy.

[45] Liu T., Ma Y., Yang W., Ji W., Wang R., Jiang P. (2022). Spatial-temporal interaction learning based two-stream network for action recognition. Inform. Sci..

